# Magnitude and predictors of pre-diabetes among adults in health facilities of Gondar city, Ethiopia: a cross-sectional study

**DOI:** 10.3389/fpubh.2023.1164729

**Published:** 2023-12-15

**Authors:** Tsegereda Abebe Andargie, Berhanu Mengistu, Lemlem Daniel Baffa, Kedir Abdela Gonete, Aysheshim Kassahun Belew

**Affiliations:** ^1^International Institute for Primary Health Care-Ethiopia, Addis Ababa, Ethiopia; ^2^Department of Human Nutrition, Institute of Public Health, University of Gondar, Gondar, Ethiopia; ^3^Caring Futures Institute, College of Nursing and Health Sciences, Flinders University, Adelaide, South Australia

**Keywords:** magnitude, pre-diabetes, predictors, adults, Ethiopia

## Abstract

**Introduction:**

Impaired glucose tolerance currently affects 374 million (7.5%) people worldwide, and by 2030, this number is predicted to affect 454 million (8%). Urban inhabitants have an increased risk of developing pre-diabetes. Thus, the study aimed to assess the magnitude of pre-diabetes and associated factors among adults attending outpatient departments of the health facilities of Gondar, Ethiopia.

**Method:**

From 3 March to 18 April 2020, an institution-based cross-sectional study was conducted. A systematic random sampling technique was used to select 992 participants. Data were gathered using an interviewer-administered questionnaire, and fasting blood glucose was assessed using capillary blood. Bivariable and multivariable binary logistic regression analyses were fitted to check the association between independent variables and pre-diabetes. Statistical significance was declared at a level of *P* of <0.05.

**Results:**

The prevalence of pre-diabetes was 16.6% (95% CIs: 14.3–18.8%). Age [AOR = 3.66, 95% CIs (2.05, 6.52)], a family history of diabetes mellitus [AOR = 3.46, 95% CIs (2.16, 5.52)], waist circumference [AOR = 3.6, 95% CIs (2.26, 5.88)], physical activity [AOR: 5.02, 95% CIs (2.87, 8.77)], dietary diversity [AOR = 3.07, 95% CIs (1.95, 4.84)], and smoking [AOR = 2.9, 95% CI (1.42, 6.05)] were factors associated with pre-diabetes.

**Conclusion:**

From our study, we can conclude that one in six adults in the health facilities have pre-diabetes. Age, family history of diabetes, waist circumference, physical activity, dietary diversity, and smoking were the factors associated with pre-diabetes. Therefore, it is recommended that adults should be educated on modifying their lifestyle, including their diet, and substantial care should be provided for older adults.

## Introduction

Pre-diabetes is characterized by a fasting blood glucose level between 100 and 125 mg/dL, postprandial blood glucose level between 140 and 199 mg/dL, or hemoglobin A1C level between 5.75% and 6.4% (1). Maintaining blood glucose levels above normal and below the recommended diagnostic threshold for diabetes is a serious public health challenge (2). Prior to the onset of type 2 diabetes, a person experiences a transitory condition known as pre-diabetes. The risk of non-communicable diseases such as diabetes, peripheral vascular disease, and cardiovascular disease increased with this condition (3, 4).

Impaired glucose tolerance affects 374 million (7.5%) individuals worldwide, and by 2030, this number is predicted to affect 454 million (8%) people (2). It is predicted that 7.9% of people with pre-diabetes will be from Africa (5). Over 2.9 million people in Ethiopia have impaired glucose tolerance and 4.9 million have diabetes. Of the 4.9 million people with diabetes, 1.4 million are yet to be identified (6).

The presence of other diseases (7); a family history of diabetes mellitus (8); sociodemographic factors, such as occupation, educational level, marital status, and urban residence (7, 9, 10); behavioral factors, such as frequent alcohol consumption (11), low levels of physical activity, poor diet diversity (12); being overweight (10, 13) or obese (14); and a history of hypertension (15) were found to be predictors of pre-diabetes.

The Ethiopian government has placed a strong emphasis on reducing the immediate and associated long-term effects of pre-diabetes by improving the quality of care and treatment for people with diabetes and raising awareness about the serious complications of diabetes by training medical personnel across the country. Individuals having diabetes are regularly monitored, and their blood glucose is managed by skilled healthcare professionals in accordance with recommended practices, which includes medical nutritional therapy (16). In contrast, people who have a higher chance of developing diabetes, such as those with impaired blood glucose, have not received much attention (11).

Furthermore, there is a lack of scientific information regarding the prevalence and determinants of pre-diabetes in various demographic groups. For instance, despite the increased susceptibility to pre-diabetes among urban inhabitants, there is scarce evidence about the magnitude and associated factors of pre-diabetes in the urban areas of Ethiopia and in other developing countries. Moreover, prevention and control of pre-diabetes has not received a due attention in these regions. Therefore, this study aimed to assess the prevalence and associated factors of pre-diabetes among adults visiting outpatient departments (OPDs) at health facilities in Gondar city, Northwest Ethiopia. Evidence of this finding can be used to design interventions aimed at reducing increased blood glucose among adults.

## Methods and materials

### Study design and setting

From 3 March to 18 April 2020, an institution based cross-sectional study was conducted at health facilities in Gondar city, Ethiopia. Gondar city is located in the Amhara region of Northwestern Ethiopia. The city is located 727 km away from Addis Ababa. According to the Ethiopian Central Statistical Agency (CSA) data of 2007, the estimated population size of the city in the study period was 391,601 persons per square mile, of which 51% were estimated to be adults (17). Currently, the city of Gondar has one referral hospital, eight health centers, and eight private clinics.

### Population, sampling procedure, and sample size determination

All adult individuals who visited adult outpatient department (OPD) clinics of health facilities in Gondar city, Ethiopia were considered to be the source population. All adults between the ages of 19 and 65 years attending adult OPDs in the selected health facilities during the data collection period were included. However, participants who were seriously ill, those who were unable to communicate, those who did not fast overnight, and pregnant and lactating mothers were excluded from this study. The sample size was determined using the single population proportion formula considering the rate of pre-diabetes among adults from a previous study (12%) (18), 3% margin of error at 95% CIs, and a design effect of 2. Therefore, after adding 10% contingency, the final sample size of the study was 992. For this study, a multistage sampling technique followed by the systematic random sampling method was used. First, three health facilities were chosen from the nine health facilities that were selected for the study by using the simple random sampling technique. Then, the sample size was proportionated to the selected health facilities. Finally, the systematic random sampling method was used to select every eight participant visiting the OPDs of the selected health facilities.

### Variables and data collection procedures

An interviewer administered the questionnaire through a face-to-face interview by implementing COVID-19 precaution measures that were used to collect the data. The questionnaire was adopted from previous similar studies with minor modifications. The questionnaire comprises sociodemographic and economic characteristics, clinical characteristics, lifestyle questions, dietary diversity, nutritional knowledge questions, and anthropometric measurements. The structured questionnaire was translated into Amharic and back to English through language and content experts. Three B.Sc. nurses and three laboratory technicians were enrolled as data collectors and supervisors, respectively. Data collectors and supervisors received 2 days of training prior to the data collection on the objective, the ethical considerations, the data collection processes, and COVID 19 safety precautions. A pre-test was done on 5% of the sample size in a similar study setting outside of the study region.

A calibrated glucometer (I-QARE) was used to measure the blood glucose of adults. The participant’s fingertip was aseptically pinched to obtain a capillary blood sample. Following the rubbing of the fingertip with a sterile cotton ball soaked in alcohol, a drop of blood was collected from the pricked finger using a sterile disposable lancet. The first drop of blood was wiped off using a sterile gauze to avoid alcohol contamination, while the second blood drop was used to assess the blood glucose and the result was confirmed within 10 s. According to the International Diabetes Federation (IDF), participants were considered to have pre-diabetes if their fasting blood glucose was between 110 and 125 mg/dL after at least 8 h of overnight fasting (19), and those who did not fall within this range for their fasting blood glucose were classified as not having pre-diabetes.

We used calibrated devices to measure both height and weight in accordance with the World Health Organization (WHO) standards. The calibrated scale was used to measure weight to the closest 100 g, and heavy clothing and shoes were taken off. Height was measured to the nearest centimeter with respondents standing in the Frankfurt position on a stadiometer. Waist circumference was measured according to the WHO recommendations to the nearest 1 cm two times at the approximate midpoint between the lower margin of the last palpable rib and the top of the iliac crest with arms at the side and feet positioned close together. However, before measuring the waist circumference, participants were asked to relax and take normal breaths to decrease the inward pull of abdominal contents. If the waist circumference was greater than 102 cm for men and 88 cm for women, then those individuals were classified as having a high risk of a metabolic problem (20).

Body mass index (BMI) is defined as the individual’s weight divided by the square of their height in meters (kg/m^2^). After calculation, a BMI of less than 18.5 kg/m^2^ is considered underweight, that of 18.5–24.9 kg/m^2^ is normal, that of 25.0–29.9 kg/m^2^ is overweight, and that of more than 30 kg/m^2^ is obese (21).

After each participant had been seated for a minimum of 10 min, blood pressure was measured using a sphygmomanometer (22) two times in 5 min intervals. Then, the average systolic blood pressure (SBP) and diastolic blood pressure (DBP) were noted. Participants were considered hypertensive if their SBP or DBP were greater than 140 mmHg or 90 mmHg, respectively (23).

The Global Physical Activity Questionnaire was used to assess the level of physical activity. It includes sixteen items divided into three domains, namely, work, travel to and from places, and recreational activities. These items also gathered the intensity (vigorous and moderate) and time of each physical activity of the participants. Based on the metabolic equivalent of task (MET)-minutes per week, participants were categorized into three levels of physical activity: sedentary physical activity (<600 MET-minutes per week), moderate physical (600–1,500 MET-minutes per week) activity, and vigorous physical activity (≥1,500 MET-minutes per week) (24).

Alcohol intake was measured by using the Fast Alcohol Screening Test (FAST) having four questions (22). A sum score of greater than or equal to 3 indicates that the participants were classified as having probable hazardous alcohol intake, whereas a sum score of less than 3 was classified as having non-probable hazardous alcohol intake (25). Participants were classified as current smoker if they have smoked at least 100 cigarettes in their lifetime, either daily or occasionally, at the time of the interview. Participants were classified as former smokers if they claimed to have smoked fewer than 100 cigarettes in their lifetime but do no longer smoke. Participants who had not smoked cigarette in their life time were classified as non-smokers (26). Khat (*Catha edulis* Forsk.) (a green leaf with a stimulant effect) chewing habits of participants were assessed according to the following: non-chewer: participants who never chewed khat, ex-chewer: participants who have chewed khat once in their lifetime, but not at least within 3 months before the start of the study, and current chewer: participants who have chewed khat within 3 months before the start of the study (27).

The dietary diversity score (DDS) was computed from ten food groups consumed within the last 24 h before the data collection. After computing the mean score, participants were categorized as having adequate DDS if they scored above or equal to the mean and inadequate DDS if they scored below the mean (28). The nutritional knowledge of the participants was measured using nine item questions that had a “Yes” or “No” response. After computing the score where “Yes” was coded as 1 and “No” was coded as 0, those whose score was greater or equal to the mean were classified as having good nutritional knowledge, while participants who scored below the mean were classified as having poor nutritional knowledge. The wealth status of the participants was computed using principal component analysis (PCA) according to the standards of demographic and health survey methodologies. Finally, the score was summed and ranked into tertiles as poor, medium, and rich (29).

### Data processing and analysis

The returned questionnaires were checked manually for completeness and consistency of the responses. Then, the cleaned data were entered into EPI INFO version 7 and the output was exported to SPSS version 20 for further analysis. Descriptive measures such as mean, frequency, and percentage were computed. A binary logistic regression analysis was conducted to examine the association between the outcome variable (pre diabetes) and each predictor variable. First, the bivariable analysis was performed to select variables for the multivariable analysis; variables with a *p*-value of less than 0.2 in the bivariable analysis were candidates for the multivariable analysis. Before running the final analysis, the assumptions of the chi-square test of independence and multicollinearity (VIF < 2) were checked. Finally, in the multivariable logistic regression analysis, variables with a *p*-value of <0.05 were considered as an independent predicator of pre-diabetes at 95% CIs. Both crude odds ratio (30) and adjusted odds ratio (31) were used to indicate the strength of the association. The fitness of the model was tested using the Hosmer-Lemeshow goodness of fit test (*p*-value = 0.9). Finally, the findings are presented using texts and tables.

## Results

### Sociodemographic characteristics of the study participants

A total of 981 subjects were enrolled in the study, yielding a 98.9% response rate. The mean age of the participants in years was 37.17 (SD ± 1 2.36). Half (50.9%) of the participants were women. More than half of the participants (57.1%) were urban residents. More than three-quarters of the participants (76.4%) were Orthodox Christians, and nearly two-thirds (61.3%) of the participants were married. With regard to educational status, more than a quarter (29%) of the participants had a certificate and above. Moreover, more than one-fifth (21.3%) of the participants were merchants, and one-third (32.9%) of the participants’ wealth status was in the poor category (Table 1).

**Table 1:** Socio demographic characteristics of adults attending outpatient departments in Gondar city health facilities, Northwest Ethiopia, 2020.

Variables	Category	Frequency	Percent
**Sex**	Female	499	50.9
	Male	482	49.1
**Age**	19–34	462	47.1
	35–44	239	24.4
	45–65	280	28.5
**Residence**	Urban	547	55.8
	Semi-urban	223	22.7
	Rural	211	21.5
**Religion**	Orthodox	749	76.4
	Muslim	177	18.0
	Protestant	47	4.8
	Catholic	8	0.8
**Ethnicity**	Amhara	899	91.6
	Tigray	34	3.5
	Other	31	3.2
	Oromo	17	1.7
**Marital status**	Married	601	61.3
	Single	263	26.8
	Divorced	62	6.3
	Widowed	32	3.3
	Separated	23	2.3
**Educational status**	Unable to read and write	140	14.3
	Able to read and write	171	17.4
	Primary	142	14.5
	Secondary	244	24.9
	Certificate and above	284	29.0
**Husband’s/wife’s educational status**	Unable to read and write	114	11.6
	Able to read and write	147	15.0
	Primary	80	8.2
	Secondary	131	13.4
	Certificate and above	152	15.5
**Occupational status**	Housewife	236	24.1
	Merchant	209	21.3
	Civil servant	173	17.6
	Private organization	140	14.3
	Student	124	12.6
	Farmer	99	10.1
**Husband’s/wife’s Occupational status**	Housewife	222	22.6
	Merchant	118	12.0
	Civil servant	118	12.0
	Farmer	81.8.3	8.3
	Private organization	72	7.3
	Student	13	1.3
**Wealth index**	Poor	323	32.9
	Medium	330	33.6
	Rich	328	33.4

### Nutritional status and clinical characteristics of the study participants

More than two-thirds of the participants (69.1%) had a normal BMI, and the large majority of the participants (82.7%) had a lower risk of metabolic complications. The vast majority (91.2%) of participants had a systolic blood pressure below 140 mmHg, and 790 (80.5%) participants had no family history of diabetes (Table 2).

**Table 2:** Nutritional status and clinical characteristics of adults attending outpatient departments in Gondar city health facilities, Northwest Ethiopia, 2020.

Variables	Category	Frequency	Percent
BMI	Underweight	41	4.2
	Normal BMI	678	69.1
	Overweight	215	21.9
	Obese	31	3.2
Waist circumference for male participants	High risk	29	3.0
	Low risk	453	46.2
Waist circumference for female participants	High risk	141	14.4
	Low risk	358	36.5
Waist circumference	High risk	170	17.3
	Low risk	811	82.7
Systolic blood pressure	Hypertensive	86	8.8
	Non-hypertensive	895	91.2
Diastolic blood pressure	Hypertensive	68	6.9
	Non-hypertensive	913	93.1
Family history of diabetes mellitus	Yes	191	19.5
	No	790	80.5
Comorbidity	Yes	57	5.8
	No	924	94.2
Type of comorbidity	Asthma	21	2.1
	Hypertension	21	2.1
	RVI	8	0.8
	Epilepsy	7	0.7
Screening for diabetes mellitus	Yes	148	15.1
	No	833	84.9
Number of screenings	Once	87	8.9
	More than once	61	6.2
Checked with in the last month	Yes	66	6.7
	No	82	8.4

### Lifestyle characteristics of the study participants

The small proportion (10.1%) of the participants had probable hazardous alcohol intake. More than two-fifth (43%) of the participants had engaged in vigorous physical activity (Table 3).

**Table 3:** Life style characteristics of adults attending outpatient departments in Gondar city health facilities, Northwest Ethiopia, 2020.

Variables	Category	Frequency	Percent
Smoking	Non-smoker	920	93.8
	Current smoker	61	6.2
Khat chewing	Non-chewer	882	89.9
	Current-chewer	50	5.1
	Ex-chewer	49	5.0
8 or more drinks (for women six or more drinks) in one occasion	Never	785	80.0
	Less than monthly	135	13.8
	Monthly	45	4.6
	Weekly	14	1.4
	Daily/almost daily	2	0.2
Not able to remember what happened because of drinking	Never	871	88.8
	Less than monthly	81	8.3
	Monthly	21	2.1
	Weekly	8	0.8
Failed to do what was expected because of drinking	Never	922	94.0
	Less than monthly	42	4.3
	Monthly	11	1.1
	Weekly	6	0.6
Concern from relative or health worker to cut down drinking	Never	904	92.2
	Yes, on one occasion	58	5.9
	Yes, more than one occasion	19	1.9
Hazardous alcohol use	<3	882	89.9
	≥3	99	10.1
Physical activity	Vigorous	422	43.0
	Moderate	317	32.3
	Sedentary	242	24.7

### Dietary knowledge and practice of the study participants

The mean score of the nutritional knowledge of the participants was 5.09 (SD ± 2.02). Similarly, almost two-thirds (60.2%) the participants had a good nutritional knowledge. In this study, almost one-third (30.9%) of the participants stated obesity as a cause of pre-diabetes. The mean score of adequate dietary diversity of the participants was 4.18 (SD ± 1.447). Accordingly, more than two-thirds (68. 4%) of the participants had adequate dietary diversity. However, only-tenth (9.3%) of the participants consume eggs and one-third (33.3%) of participants consume dark leafy vegetables.

### Prevalence and associated factors of pre-diabetes among study participants

The prevalence of pre-diabetes was 16% (95% CIs, 14.3, 18.8%). Moreover, age, residence occupation, family history of diabetes mellitus, body mass index, systolic blood pressure, diastolic blood pressure, waist circumference, smoking dietary diversity, and physical activity were associated with pre-diabetes in the bivariable logistic regression. However, multivariable regression results revealed that age, family history of diabetes, waist circumference, smoking, physical activity and dietary diversity were significantly associated with pre-diabetes.

The odds of having pre-diabetes was 3.66 [AOR: 3.66 95% CIs (2.05, 6.52)] and 2.4 [AOR: 2.41 95% CIs (1.31, 4.4)] times higher among older adults and middle-aged adults, respectively, when compared to their young counterparts. Participants with a family history of diabetes had a 3.46 [AOR: 3.46, 95% CI (2.16, 5.527)] times higher odds of having pre-diabetes when compared to participants without a family history of diabetes. The odds of having pre-diabetes was 3.65 [AOR: 3.65, 95% CIs (2.26–5.88)] times higher among participants whose waist circumference was at high risk when compared to participants with waist circumference at the low risk. Besides, the odds of having pre-diabetes was three [AOR: 3.08, 95%CIs (1.95–4.84)] times higher among participants with inadequate dietary diversity when compared to participants with adequate diet diversity. Moreover, compared with participants who exercised vigorously, participants who engaged in a low level of exercise had 5 [AOR: 5.02, 95% CIs (2.87–8.77)] times higher odds of having pre-diabetes. Finally, smokers had 2.9 [AOR: 2.95%, CIs (1.42–6.05)] times higher odds of having pre-diabetes when compared to their counterparts (Table 4).

**Table 4:** Factors associated with prediabetes among adults attending outpatient departments in Gondar city health facilities, Northwest Ethiopia 2020.

Variables	Pre-diabetes		COR (95% CI)	AOR (95% CI)
	Yes	No		
**Age (years)**				
19-34 (Young adult)	28 (6.1)	434 (93.9)	1	1
35-44 (Mid aged adult)	45 (18.8)	194 (81.2)	3.59 (2.17, 5.93)	2.42 (1.31, 4.44) *
45-65 (Old adult)	90 (32.1)	190 (67.9)	7.34 (4.65, 11.59)	3.66 (2.056, 6.52) *
**Residence**				
Urban	100 (18.3)	447 (81.7)	1.83 (1.13, 2.96)	0.771 (0.35, 1.78)
Semi-urban	40 (17.9)	183 (82.1)	1.78 (1.02, 3.10)	0.53 (0.22, 1.26)
Rural	23 (10.9)	188 (89.1)	1	1
**Occupation**				
Housewife	43 (18.2)	193 (81.8)	1.13 (0.65, 1.97)	0.9 (0.45, 1.82)
Farmer	11 (11.1)	88 (88.9)	0.63 (0.29, 1.37)	0.91 (0.3, 2.75)
Student	4 (3.2)	120 (96.8)	0.17 (0.06, 0.5)	0.38 (0.11, 1.32)
Merchant	48 (23.0)	161 (77.0)	1.52 (0.87, 2.63)	1.07 (0.55, 2.09)
Civil servant	34 (19.7)	139 (80.3)	1.24 (0.69, 2.23)	0.79 (0.38, 1.63)
Private organization	23 (16.4)	117 (83.6)	1	1
**BMI**				
18.5–24.9	74 (10.9)	604 (89.1)	1	1
<18.5	3 (7.3)	38 (92.7)	0.64 (0.19, 2.14)	1.18 (0.33, 4.28)
25.0–29.9	67 (31.2)	148 (68.8)	3.69 (2.53, 5.38)	1.17 (0.73, 1.89)
>30	13 (41.9)	18 (58.1)	5.89 (2.77, 12.5)	1.76 (0.66, 4.66)
**Waist circumference**				
High risk	71 (41.8)	99 (58.2)	5.61 (3.85, 8.15)	3.65 (2.26, 5.88) *
Low risk	92 (11.3)	719 (88.7)	1	1
**Systolic blood pressure**				
Hypertensive	33 (38.4)	53 (61.6)	3.66 (2.28, 5.87)	1.69 (0.92, 3.13)
Non-hypertensive	130 (14.5)	765 (85.5)	1	1
**Diastolic blood pressure**				
Hypertensive	19 (27.9)	49 (72.1)	2.07 (1.18, 3.62)	1.08 (0.52, 2.24)
Non-hypertensive	144 (15.8)	769 (84.2)	1	1
**Family history**				
Yes	69 (36.1)	122 (63.9)	4.19 (2.91, 6.04)	3.46 (2.17, 5.53) *
No	94 (11.9)	696 (88.1)	1	1
**Smoking**				
Yes	19 (31.1)	42 (68.9)	2.44 (1.37, 4.31)	2.93 (1.42, 6.05)
No	144 (15.7)	776 (84.3)	1	1
**Physical activity**				
Sedentary	87 (36.0)	155 (64.0)	6.02 (3.912, 9.26)	5.02 (2.87, 8.77) *
Moderate	40 (12.6)	277 (87.4)	1.54 (0.96, 2.49)	1.48 (0.82, 2.67)
Vigorous	36 (8.5)	386 (91.5)	1	1
**Nutritional knowledge**				
Good	90 (15.2)	501 (84.8)	1	1
Poor	73 (18.7)	317 (81.3)	1.28 (0.91, 1.80)	1.03 (0.66, 1.63)
**Dietary diversity**				
Adequate	88 (13.1)	583 (86.9)	1	1
Poor	75 (24.2)	235 (75.8)	2.11 (1.5, 2.98)	3.07 (1.95, 4.84) *

*Indicate *p*-value < 0.05.

## Discussion

Pre-diabetes denotes a heightened risk of non-communicable diseases. In cognizant of this, our study sought to examine the prevalence and predictors of pre-diabetes among adults attending the OPDs of the health facilities in Gondar city, Ethiopia.

Therefore, our findings revealed that one in six [16.6% (95% CIs: 14.3, 18.8%)] participants of the adults had pre-diabetes. This finding is in line with the reports from India (32), Saudi Arabia (33), and Ethiopia (34), with a prevalence of pre-diabetes among adults of 15.4%, 17.3%, and 15.9%, respectively. The study design and having a similar study population might be the possible explanation. Furthermore, being an institution-based study, we could explain the similarity of our findings with the report from Saudi Arabia.

The prevalence of pre-diabetes among adults in the current study is higher than that on the reports from Vietnam (8.1%) (8), India (6.3%) (35), Omani (9%) (36), Saudi Arabia (6.8%) (37), Uganda (8.6%) (12), Cameron(5.7%) (38), Egypt (7.3%) (7), and Kenya (12%) (9) and pocket area studies in Ethiopia (8%–12.9%) (10, 18, 39, 40), which could be due to differences in sample size and study settings. Moreover, the living conditions could explain this discrepancy. For instance, participants from Egypt, Saudi Arabia, and India (7, 35, 37) had a better income status than our study participants, which in turn improved their access to a better health service, including getting nutritional counseling (41). However, comparing this study with other studies in Ethiopia, people residing in urban areas are experiencing poor lifestyle changes, which includes an increased consumption of high energy but low quality foods and living a sedentary lifestyle (42).

However, the prevalence of pre-diabetes of the current study is lower than the studies in China (19.8%–28.5%) (13, 43) and Saudi Arabia (23.6%) (44) and pocket area studies in Ethiopia (20.3%–22.4%) (11, 45), which might be explained by urbanization in China and Saudi Arabia, which has a higher impact on causing a sedentary lifestyle, rapid nutrition transition, and also a variation in dietary habits among the population (46). The mean age of the participants being lower in our study could explain this discrepancy from a previous study in Ethiopia (47).

The study revealed that older adults had higher odds of developing pre-diabetes when they were compared to young adults. This finding is consistent with studies conducted in China (13), Egypt (7) and Cameroon (38). This possible explanation may be because, with age, β cells become insufficient to produce insulin and the sensitivity of human cells to insulin decreases (48). In addition, the older adults tend to engage in less physical activity, which in turn makes them prone to complications that may have a profound effect on blood glucose (49).

Compared with their peers, participants with a higher waist circumference had higher odds of developing pre-diabetes. This is supported by a cross-sectional study in northeastern China (13). Studies have showed that higher waist circumference could lead to the accumulation of adipose tissues that results in insulin resistance, which subsequently causes pre-diabetes and type 2 diabetes mellitus (49). Moreover, obesity is linked with inflammatory cytokines such as tumor necrosis factor-alpha (TNF-α), interleukin-6 (IL-6), and monocyte chemoattractant protein 1 (MCP-1), which are particularly higher among individuals with central obesity. An elevated amount of adipocytes and the increased production of TNF-α and IL 6 induce insulin resistance (50, 51). Adults with a family history of diabetes are 3.4 times more likely to develop diabetes than their counterparts. This finding was supported by studies in China (52) and Vietnam (8). Genetic anomalies in pancreatic beta cells and the chemicals that make up the molecular mechanism of insulin action could be one explanation (53). In addition, a well-known risk factor for early-onset diabetes and associated consequences is a family history of diabetes (54).

The finding of this study has also revealed that having a low level of physical activity increases the odds of pre-diabetes. This finding was supported by studies conducted in China (13), Saudi Arabia (33), and Kenya (9). This association could be explained by decreased glucose uptake at rest, which resulted from decreased skeletal muscle contraction. As a person’s level of physical activity declines, so does the impact of insulin on their muscles and liver (55).

In this study, smokers had higher odds of developing pre-diabetes than non-smokers. This result is supported by a finding from Southern India (56). One theory is that nicotine causes skeletal muscle to become insulin resistant, which results in pancreatic beta-cell apoptosis and decreased beta-cell mass (57). Furthermore, smoking causes nicotine-induced fat breakdown that increases the re-esterification of free fatty acids and increases the production of very low-density lipoprotein in the liver that promotes the formation of fat deposits in arteries, providing the opportunity for other risk factors such as obesity to cause pre-diabetes (58).

Moreover, adults with insufficient dietary diversity had higher odds of getting pre-diabetes than those with adequate dietary diversity. This result is comparable to studies conducted in Uganda (12). This result can be explained by the fact that individuals who consume a homogenous diet are more prone to have hyperlipidemia and other metabolic risk factors, such as central obesity, which is also brought on by a sedentary lifestyle that results in insulin resistance (59).

This study has several limitations, of which one involves not investigating the level of dyslipidemia, which may have influenced participants’ blood glucose. As the current study was an institution-based cross-sectional study, the prevalence of pre-diabetes might have been overestimated. In addition, we were unable to establish a causal link between the predictor and the outcome variables because of the cross-sectional nature of the study design. The use of a single diagnostic modality and fasting blood glucose, but not using glucose tolerance test (GTT) and HbA1c might result in missing of individuals with impaired glucose tolerance (IGT). Moreover, unknown thyroid status of the participants that might have an impact on glucose measurement should not be overlooked, which is another limitation of the current study.

### Implication of the study

The finding of the current study implies the emerging unhealthy dietary practices and sedentary lifestyles in developing countries are being an entry point for non-communicable diseases. Besides, being old and central obesity were also expected to increase the magnitude of pre-diabetes. Therefore, the findings of this study imply the importance of the urgent need of identifying individuals with an increased risk of type 2 diabetes mellitus to halt the emerging diet related non-communicable diseases in developing countries.

## Conclusion

In our study, we found that one in six adults in the health facilities have pre-diabetes. Age (being old), having a family history of diabetes, increased waist circumference, low level of physical activity, poor dietary diversity, and smoking are factors related to pre-diabetes. Therefore, special efforts targeted at education on changing one’s lifestyle, toward special care for older adults, and at improved food security for diet diversification are highly recommended.

## Data availability statement

The raw data supporting the conclusions of this article will be made available by the authors, without undue reservation.

## Ethics statement

The studies involving humans were approved by Institutional Review Board (IRB) of Institute of Public Health, University of Gondar. The studies were conducted in accordance with the local legislation and institutional requirements. The participants provided their written informed consent to participate in this study.

## Author contributions

TA, AB, and KG developed concepts and designs of study. TA, AB, KG, LB, and BM supervised data collection and ensured data quality. TA and BM have cleaned and analyzed the data as well as interpreted the results and drafted the manuscript. AB, KG, LB, and BM critically revised the manuscript. All authors contributed to the article and approved the submitted version.
